# Iron-related biomarkers and Lewy body dementia: a scoping review

**DOI:** 10.3389/fnagi.2026.1900516

**Published:** 2026-10-08

**Authors:** Jessica L. Freeling, Lisa M. McFadden

**Affiliations:** 1 FreelingBio Research Consulting, LLC, Vermillion, SD, United States; 2 Biomedical and Translational Science, University of South Dakota, Vermillion, SD, United States

**Keywords:** Lewy body dementia, dementia with Lewy bodies, Parkinson’s disease dementia, iron dysregulation, brain iron, biomarkers

## Abstract

**Background and Aims:**

Iron dysregulation is implicated in neurodegenerative disease pathophysiology, but its role in Lewy body dementia (LBD) remains poorly characterized. This review mapped iron-related measures across blood, cerebrospinal fluid (CSF), and brain compartments and evaluate their potential relevance as biomarkers in LBD.

**Methods:**

A scoping review was conducted according to PRISMA-ScR guidelines using PubMed and Web of Science. Studies reporting iron-related biomarkers in individuals with LBD were eligible regardless of biological compartment; review articles and non-LBD studies were excluded. Of 222 identified records, 24 met inclusion criteria. Data were charted using a standardized extraction framework and due to methodological heterogeneity, were synthesized qualitatively across blood and CSF measures, postmortem brain analyses, and *in vivo* neuroimaging.

**Results:**

Brain-based measures showed the most consistent iron-related alterations, particularly quantitative susceptibility mapping (QSM), with abnormalities across cortical, limbic, and subcortical regions, although findings were not universal. Susceptibility-weighted imaging (SWI) provided complementary evidence of nigrosome-1 abnormalities. Peripheral and CSF measures were heterogeneous and showed no consistent directional pattern. No study directly compared measures across all three compartments within the same cohort.

**Conclusion:**

Iron dysregulation in LBD appears more pronounced in brain than systemic compartments, though within-cohort cross-compartment comparisons are lacking. Brain iron accumulation extends beyond the substantia nigra, aligning with Lewy body pathology and potentially explaining LBD’s distinctive cognitive and neuropsychiatric features. QSM and SWI are promising candidate imaging biomarkers, though current evidence reflects group-level differences rather than individual-patient diagnostic accuracy. Longitudinal, cross-compartment studies are needed to clarify their clinical significance in LBD.

## 1 Introduction

Iron is fundamental to brain function, supporting mitochondrial energy production (Dietz et al., 2021), DNA synthesis and repair (Puig et al., 2017), and neurotransmitter synthesis and myelination (Hare et al., 2013), while remaining tightly regulated to limit oxidative stress (Singh et al., 2014; Levi et al., 2024; Chen et al., 2025). Even under normal conditions, brain iron distribution varies by region with age-related accumulation concentrated in the substantia nigra (SN) and other dopaminergic circuits (Ward et al., 2014; Gao et al., 2025). This regional variability underscores why iron dysregulation may have distinct consequences across brain regions. In neurodegenerative diseases, the location and timing of neuronal injury may ultimately determine clinical presentation.

Iron homeostasis operates through distinct regulatory processes in each biological compartment, producing measurable iron pools that reflect different aspects of iron biology (Chifman et al., 2014). Blood measures primarily capture peripheral iron availability and hematologic balance (Garcia-Casal et al., 2021), whereas cerebrospinal fluid (CSF) measures reflect iron transport, buffering, and exchange at the blood brain interface (Moos and Morgan, 2000; Zheng and Monnot, 2012). In contrast, brain iron reflects regional uptake, storage, and cellular handling (Ward et al., 2014), driven by local metabolic demand rather than systemic iron status (Cheli et al., 2020). These compartments are regulated partly independently, and this separation has direct implications for how iron-related findings should be interpreted in neurological disease research.

Lewy body dementia (LBD) is a collective term encompassing two closely related clinical syndromes: dementia with Lewy bodies (DLB) and Parkinson’s disease dementia (PDD) (Galvin, 2024). Both share underlying α-synuclein pathology but are distinguished clinically by timing of cognitive and motor symptom onset. In DLB, dementia precedes or coincides with parkinsonian motor features, whereas in PDD, motor symptoms are established for at least 1 year before dementia onset (McKeith et al., 2017). This phenotypic variability likely reflects differences in pathology distribution rather than uniform disease progression. Core clinical features include fluctuating cognition, recurrent visual hallucinations, Rapid eye movement sleep behavior disorder (RBD), and parkinsonism, with additional variability across autonomic and neuropsychiatric domains (McKeith et al., 2017; Kaila et al., 2025). This clinical complexity contributes to underdiagnosis and misclassification, with diagnostic delays that carry consequences for patients and caregivers (Armstrong and Barnes, 2024; Galvin, 2024). Reliable biomarkers remain an unmet need.

Iron dysregulation in Parkinson’s disease (PD) is well-studied, with regional brain iron accumulation a relatively consistent finding (Sian-Hülsmann et al., 2011; Ma et al., 2021). However, the relevance to LBD remains unclear. Studies span multiple biological compartments and methodological approaches. Whether iron dysregulation in LBD reflects mechanisms shared with PD, distinct regulatory processes, or methodological variation remains an open question. To address this question, a scoping review was preformed given the heterogeneity of available evidence, spanning biological compartments, experimental designs, and outcome measures, precludes quantitative synthesis. The objective of this review is to map iron-related evidence across blood, CSF, and brain compartments in individuals with LBD, including DLB and PDD, by applying a compartment-specific framework to assess the potential of iron-sensitive measures as clinically informative biomarkers.

## 2 Methods

### 2.1 Databases and search strategy

This scoping review was conducted and reported in accordance with the Preferred Reporting Items for Systematic Reviews and Meta-Analyses extension for Scoping Reviews (PRISMA-ScR) guidelines. A review protocol was not prospectively registered given the exploratory nature of the review. A structured literature search was conducted using MEDLINE (via PubMed) and Web of Science (Core Collection). Searches were performed without date restrictions to capture the full scope of available literature and last executed on September 13, 2026. Because iron is regulated differently across biological compartments, search terms spanning systemic, CSF, and brain measures were included to capture complementary aspects of iron biology. Database-specific search strategies incorporated controlled vocabulary terms of iron-related biomarkers and keyword combinations relevant to LBD, DLB, PDD, or related clinical and prodromal phenotypes. Studies that defined Lewy body ‘disease’ based on neuropathological criteria were also captured.

Search terms were designed to be broad and compartment-agnostic (e.g., ‘iron,’ ‘ferritin,’ ‘Lewy body dementia’) rather than modality-specific, to avoid biasing retrieval toward any single biological compartment or measurement approach. Following peer review, we additionally re-ran the search in both databases with modality-specific imaging terms (quantitative susceptibility mapping, QSM, susceptibility-weighted imaging, SWI, R two-star, R2*, nigrosome-1, dorsolateral nigral hyperintensity), identifying additional eligible studies which were incorporated into this review. Full search strategies for each database are provided in Supplementary material 1. Terminology for Lewy body diseases is not standardized across the literature; studies vary in their use of DLB, PDD, and related labels, and diagnostic or pathological overlap with Alzheimer’s disease (AD) and other dementias are common. We use ‘LBD’ as an umbrella term throughout this review, while preserving study-specific terminology (e.g., DLB, PDD) when describing individual included studies and their original diagnosis.

### 2.2 Inclusion criteria

Studies were eligible for inclusion if they examined LBD–relevant phenotypes, including DLB or PDD, and reported at least one iron-related biomarker or iron-inherent imaging method measured in a relevant biological compartment. The search was not restricted by species; some animal studies were identified through the search but were excluded during screening. All eligible studies involved human participants or tissue. Reported biomarkers spanned systemic measures (e.g., ferritin, transferrin, serum iron, soluble transferrin receptor, and hemoglobin), CSF measures (e.g., ferritin, iron, or transferrin), and brain measures. Brain iron measures included *in vivo* neuroimaging techniques, such as magnetic resonance imaging (MRI) quantitative susceptibility mapping (QSM), which estimates tissue magnetic susceptibility, as well as R2* and susceptibility-weighted imaging, in addition to postmortem assessments of brain iron. This list reflects the types of iron-related measures identified in the literature and was not used to restrict inclusion. Observational, imaging, postmortem, and experimental study designs were eligible for inclusion.

### 2.3 Exclusion criteria

Studies were excluded if they did not report iron-related blood or CSF measures, iron-related imaging measures, or focused on neurodegenerative disorders other than LBD, e.g., PD without dementia. Review articles, meta-analyses, and case-studies addressing iron in LBD were retained for background and contextual reference only and were not included in the primary literature synthesis. *In vitro* studies lacking direct relevance to human physiology were excluded, as well as animal studies. Screening was not restricted by language; however, only studies with an available English full text were included in the final synthesis. Non-English full-text articles were excluded, as translation was not performed.

### 2.4 Screening, data extraction, and charting

All records retrieved from the database searches were imported into Rayyan and screened in a two-stage process. Titles and abstracts were independently screened by two reviewers, and full texts of all potentially eligible articles were independently assessed by both reviewers. Discrepancies were resolved by discussion. Relevant content was extracted from all included studies by one reviewer using a standardized charting framework and summarized in table form. Extracted variables included study design and sample size, biomarkers measured, biological compartment assessed, diagnostic criteria used for LBD, and key iron-related findings. Charted data were independently verified by a second reviewer, and interpretations were developed through discussion to ensure consistency. No contact with study investigators was made to confirm or supplement extracted data. Forward and backward citation searching of included studies was not systematically performed. Articles identified outside the formal database search were considered candidates for inclusion and screened against the same eligibility criteria.

### 2.5 Methodological considerations

Included studies were assessed narratively for methodological quality, considering study design, sample characteristics, diagnostic criteria, comparator groups, and analytic approach. No formal predefined risk-of-bias tools were applied, consistent with scoping review methodology, which prioritizes breadth of evidence mapping over quality-based exclusion. Given the heterogeneity spanning study designs, brain regions assessed, imaging and biochemical metrics used, patient populations (DLB, PDD, or combined LBD cohorts), acquisition protocols, reported outcome measures, and tissue compartment sources, a narrative synthesis was deemed the appropriate approach. Appraisal findings are presented within the strengths and limitations subsections accompanying each biological compartment section and summarized in Supplementary Table 1. This heterogeneity also precluded quantitative synthesis and confirmed the appropriateness of a scoping rather than systematic review design. The review was conducted according to an internal protocol that was not formally registered in a public registry.

## 3 Results

### 3.1 Article characteristics

As shown in Figure 1, database searches and outside record discovery identified 222 records. After removal of duplicates, 103 unique records were screened. Of these, 79 records were excluded, and 24 studies met the predefined inclusion criteria.

**Figure 1:**
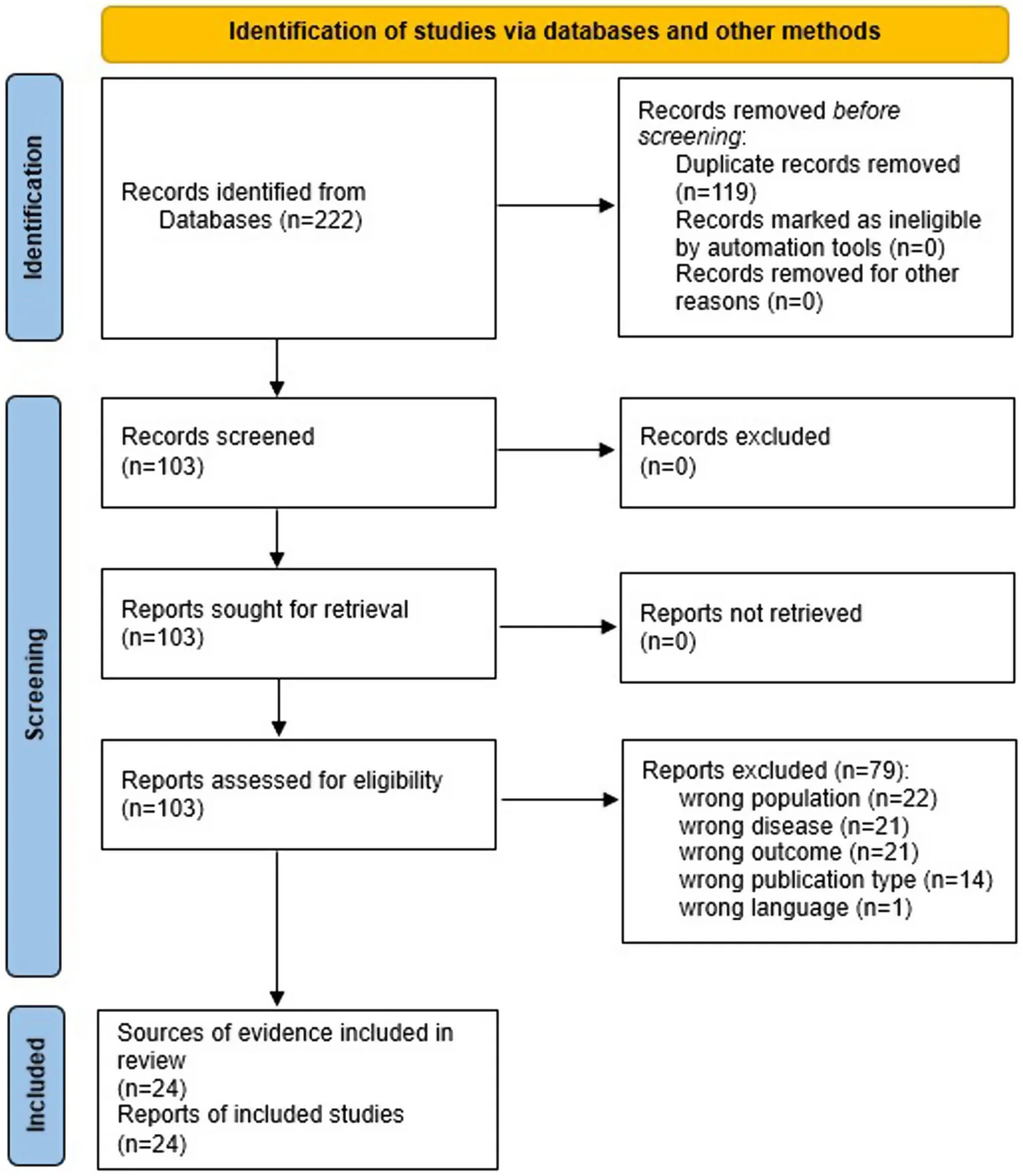
PRISMA-ScR flow diagram for scoping reviews.

### 3.2 Study characteristics

The 24 included studies (Gu et al., 1998; Magaki et al., 2007; Boström et al., 2009; Akatsu et al., 2012; Reuck et al., 2014; Streit and Xue, 2016; Kamagata et al., 2017; Shams et al., 2017; Li et al., 2018; Rizzo et al., 2018; Chen et al., 2021; Scholefield et al., 2021, 2024; Ayton et al., 2022; Chabran et al., 2022; Bhome et al., 2025; Cook et al., 2025; Diaz-Galvan et al., 2025; Karismaz et al., 2025; Ren et al., 2025; Urbano et al., 2025; Capogna et al., 2026; Guo et al., 2026; Musaeus et al., 2026), comprised a diverse body of evidence and primarily consisted of retrospective or cross-sectional observational designs. Of the 24 studies, 7 investigated peripheral iron-related markers, 7 employed postmortem brain analysis methods, and 10 utilized *in vivo* neuroimaging approaches. Peripheral iron studies examined blood or CSF markers related to iron handling, whereas postmortem studies primarily evaluated metal concentrations and imaging in brain tissue. The included studies spanned publication years from 1998 to 2026 and were conducted across multiple geographic regions. Variation in study design, populations, and iron-related outcomes precluded quantitative synthesis. Key characteristics of the included studies are summarized in Supplementary Table 1.

### 3.3 Peripheral iron measures

Seven of the 24 included studies evaluated iron-related measures in blood, CSF, or both compartments in individuals with LBD (Boström et al., 2009; Ayton et al., 2022; Cook et al., 2025; Karismaz et al., 2025; Ren et al., 2025; Urbano et al., 2025; Guo et al., 2026). Assessments primarily included hematologic indices and circulating markers of iron status (Boström et al., 2009; Cook et al., 2025; Karismaz et al., 2025; Ren et al., 2025; Guo et al., 2026), such as hemoglobin, serum iron, ferritin, transferrin, and related parameters. CSF-based assessments (Boström et al., 2009; Ayton et al., 2022; Cook et al., 2025; Urbano et al., 2025) most commonly examined ferritin and iron concentrations, with select studies also evaluating iron transport or binding proteins, such as transferrin.

Findings in CSF were primarily cross-sectional and observational. Among studies that explicitly reported CSF iron-related measures, one reported a statistically significant decrease in CSF transferrin (Cook et al., 2025), one reported no difference in CSF ferritin (Ayton et al., 2022), and another no difference in CSF iron (Boström et al., 2009) relative to controls. Despite no group differences, higher ferritin levels were associated with worse cognitive performance in a combined DLB/PDD cohort. A single prospective CSF study (Urbano et al., 2025) enrolled subjects at onset of mild cognitive impairment and followed them to various dementia diagnoses including LBD. Higher inorganic iron was associated with increased risk of conversion to dementia. However, only two participants progressed to LBD, preventing statistical conclusions specific to LBD.

Studies utilizing iron-related blood metrics were similarly variable (Boström et al., 2009; Cook et al., 2025; Karismaz et al., 2025; Ren et al., 2025; Guo et al., 2026). One study reported decreased plasma iron (Boström et al., 2009), another reported no difference in serum transferrin relative to controls after covariate adjustment (Cook et al., 2025) and another (Ren et al., 2025) reported increased serum ferritin relative to control subjects. Another study reported decreased serum transferrin in PDD patients compared to PD patients without dementia (Guo et al., 2026). Similarly, a single study (Karismaz et al., 2025) explicitly evaluated anemia prevalence, reporting clinically relevant anemia in 46.9% of patients with DLB; however, the absence of a healthy control group limits interpretation. Within this cohort, anemia was associated with a higher prevalence of depressive symptoms and greater number of medications. In two studies, higher serum ferritin levels were associated with worse cognitive performance (Ayton et al., 2022; Ren et al., 2025).

Two studies reported both blood and CSF measures within the same study population (Boström et al., 2009; Cook et al., 2025). Cook et al. (2025) found significant differences confined to the CSF in transferrin, whereas Boström et al. (2009) reported significant differences in iron restricted to the blood.

#### 3.3.1 Strengths and limitations of peripheral studies

Strengths include the use of clinically accessible markers such as ferritin and transferrin, with several studies incorporating comprehensive laboratory panels, well-characterized cohorts, and adjustment for key covariates such as age and sex. Some studies also examined associations with clinical symptoms, including cognition.

Limitations include the predominance of cross-sectional designs, modest sample sizes, and inconsistent cohort definitions, including pooling of DLB and PDD. Iron-related blood and CSF metrics also capture distinct aspects of iron biology. Serum/CSF iron concentration reflects circulating iron at the time of sampling, ferritin reflects intracellular storage, and is also an acute-phase reactant, and transferrin reflects iron-binding and transport capacity (Wish, 2006). Only one of the included studies reported transferrin saturation (Guo et al., 2026) but did not include a healthy control group, which may have offered additional mechanistic information beyond isolated iron or transferrin measures. Findings for one metric should therefore not be assumed to generalize to another, even within the same compartment. Adjustment for disease duration and severity was variable, and cognitive measures were not uniformly incorporated. Biomarker selection and analytic approaches varied widely, and circulating markers may reflect systemic influences such as inflammation, comorbidities, or medication use rather than central iron biology (Garcia-Casal et al., 2021; Charlebois and Pantopoulos, 2023). Key modifiers of iron status, including inflammatory markers and measures such as soluble transferrin receptor, were not consistently assessed (Infusino et al., 2012; Fairweather-Tait et al., 2014). CSF measures, while anatomically proximal, remain indirect, which limits cross-compartment comparisons (Moos and Morgan, 2000; Zheng and Monnot, 2012).

Together, peripheral iron measures show variable findings without a consistent direction of change, limiting their ability to define a clear pattern of iron dysregulation in LBD. However, several studies reported associations between iron markers and clinical LBD features, suggesting that, despite inconsistent group-level findings, peripheral measures may capture clinically meaningful variation within LBD.

### 3.4 Postmortem brain iron and tissue-based evidence

Seven of the 24 included studies evaluated iron in postmortem brain tissue from individuals with LBD (Gu et al., 1998; Magaki et al., 2007; Akatsu et al., 2012; Reuck et al., 2014; Streit and Xue, 2016; Scholefield et al., 2021, 2024). All were cross-sectional and observational and brain regions examined varied across studies. Approaches included quantitative spectroscopy methods, semi-quantitative histochemical staining, and semi-quantitative postmortem MRI supported analysis.

Across studies, findings were mixed: several reported increased iron concentrations in specific regions, whereas others found no significant differences in the same or other regions of DLB brains relative to controls. Among the quantitative spectroscopy studies demonstrating increased iron, increases in iron were found in the cingulate cortex (Gu et al., 1998), frontal gray matter (Magaki et al., 2007), and motor cortex and cingulate gyrus (Scholefield et al., 2024). Another spectroscopy study (Akatsu et al., 2012) reported an approximate 10% increase in iron within the hippocampus and amygdala, although this did not reach statistical significance. The remaining quantitative spectroscopy study found no difference, however, only included the brains of patients with PDD and controls were mismatched in age. Utilizing semi-quantitative ferritin immunohistochemistry, no increase in ferritin-positive microglia were found (Streit and Xue, 2016), but other cell types were not investigated. Finally, utilizing semi-quantitative MRI iron scores (R0–R3 scale), no differences in regional iron burden were found (Reuck et al., 2014).

#### 3.4.1 Strengths and limitations of postmortem studies

Strengths include the use of quantitative approaches, particularly mass spectrometry–based methods, allowing direct measurement of iron across multiple brain regions. Several studies incorporated careful tissue handling procedures, including correction for postmortem delay and tissue mass, and contamination control. Neuropathological confirmation of diagnosis was common, and several studies included age and sex matched control groups. A subset of studies examined multiple brain regions or distinct iron pools and applied region specific or multivariate analyses, supporting more comprehensive characterization of brain iron distribution.

Limitations include the cross-sectional nature of all studies, including likely end-stage disease, precluding assessment of progression. Sample sizes were often small and varied across regions, limiting statistical power. Brain region selection and analytic approaches differed across studies, with many relying on bulk tissue measurements or total iron without distinguishing iron pools. Cohort characterization was inconsistent, with some studies including variable control matching and age differences between groups, an important consideration given age-related iron accumulation (Ward et al., 2014; Gao et al., 2025). Clinical phenotyping was often limited, and some studies combined diagnostic groups or relied solely on neuropathological classification. Adjustment for disease duration, severity, and comorbid pathology was inconsistent, and coexisting AD pathology was present in several cohorts. Variability in postmortem interval, tissue preservation, and fixation methods may also influence measured iron concentrations (Pfefferbaum et al., 2004; Schrag et al., 2010; Hametner et al., 2018; Yao et al., 2025), while semi-quantitative approaches further limit precision.

Taken together, postmortem quantitative studies suggest alterations in brain iron with a directional signal toward increased iron in select regions, despite variability across studies. However, small sample sizes and methodological variability, including differences in cohort characteristics such as age, likely contribute to inconsistencies of significant findings across studies rather than the absence of a biological signal.

### 3.5 *In vivo* neuroimaging evidence of brain iron alterations

Ten of the 24 studies investigated brain iron in LBD using *in vivo* neuroimaging approaches. QSM was the most frequently employed modality used in 6 studies (Li et al., 2018; Chen et al., 2021; Bhome et al., 2025; Diaz-Galvan et al., 2025; Capogna et al., 2026; Musaeus et al., 2026), three studies employed SWI (Kamagata et al., 2017; Shams et al., 2017; Rizzo et al., 2018), and one study used the quantitative MRI-based R2* metric (Chabran et al., 2022). Across imaging modalities, susceptibility measures were interpreted as proxies for iron-related alteration, although findings differed by technique and, within QSM specifically, by region and cohort composition.

Of the six QSM studies, five consistently demonstrated increased QSM-derived susceptibility values in individuals with LBD compared with healthy or cognitively impaired controls (Li et al., 2018; Chen et al., 2021; Bhome et al., 2025; Diaz-Galvan et al., 2025; Capogna et al., 2026). Although the specific regions varied, findings spanned both cortical and subcortical structures, including the hippocampus, frontal and temporal cortices, basal ganglia, thalamus, and brainstem, and in three studies, the SN (Chen et al., 2021; Diaz-Galvan et al., 2025; Capogna et al., 2026).

Three studies investigated changes in patients who met the full criteria for LBD, whether by clinical diagnosis or neuropathological confirmation. Bhome et al. (2025) reported increased QSM-derived susceptibility values across multiple brain regions including the bilateral superior frontal, bilateral medial temporal, left superior temporal, right precentral and left cerebellar cortex in individuals with effects maintained after correction for total brain volume. Further, QSM-derived susceptibility values were positively correlated with overall disease severity in DLB as measured by total Movement Disorder Society-Unified Parkinson’s Disease Rating Scale (MDS-UPDRS). Similarly, Li et al. (2018) observed increased susceptibility in the bilateral hippocampus in PDD compared with controls, with higher signal in the left hippocampus relative to PD without dementia. Moreover, hippocampal susceptibility correlated with Mini-Mental State Examination (MMSE) scores. Notably, Diaz-Galvan et al. (2025) is the only *in vivo* QSM study to directly confirm case status through neuropathological examination at autopsy, rather than relying on clinical diagnosis alone. Higher QSM values in the SN pars compacta distinguished individuals with confirmed Lewy-related pathology from those without.

Capogna et al. (2026) similarly reported widespread increases in QSM-derived susceptibility in DLB relative to cognitively unimpaired older adults, spanning the bilateral SN, globus pallidus, thalamus, brainstem, and hippocampus, as well as posterior cortical and orbitofrontal regions. Susceptibility values were not significantly associated with brain volume, symptom duration, or cognitive performance; however, regional QSM patterns distinguished DLB patients by their earliest clinical presentation (cognitive/psychiatric-predominant versus REM sleep behavior disorder/motor-predominant onset), suggesting that regional QSM-derived susceptibility measures may relate to phenotypic heterogeneity within DLB.

Chen et al. (2021) extended these findings across the disease spectrum, demonstrating progressively increased QSM-derived susceptibility values in the SN from controls to prodromal synucleinopathy to probable DLB. This gradient suggests that iron-related changes may emerge early and continue to evolve throughout disease progression, although no associations were observed with motor or cognitive measures in this cohort.

In contrast, Musaeus et al. (2026) found no significant group differences in QSM-derived susceptibility across the hippocampus, pallidum, caudate, or putamen when comparing DLB, AD, and cognitively healthy controls, nor in the SN specifically. A significant difference was observed in the nucleus ruber, with higher QSM-derived susceptibility values in DLB relative to the other groups. These findings indicate that increased QSM-derived susceptibility values in LBD are not uniformly detected across all subcortical structures and may be sensitive to sample size, region selection, and acquisition parameters.

Three studies used SWI to assess the dorsolateral nigral hyperintensity, or “swallow tail sign,” a susceptibility-sensitive marker of nigrosome-1 integrity distinct from the quantitative tissue susceptibility values derived using QSM. Kamagata et al. (2017) combined visual assessment with a semi-quantitative nigrosome-1 contrast ratio and found reduced nigrosome-1 signal in DLB relative to healthy controls and individuals with AD or amnestic mild cognitive impairment (a-MCI); the contrast ratio demonstrated high sensitivity and specificity for distinguishing DLB from AD/a-MCI. Shams et al. (2017) similarly found that an abnormal swallow tail sign was significantly more common in LBD than in other diagnostic groups and controls, although diagnostic performance varied by rater and imaging parameters. Finally, Rizzo et al. (2018) subsequently reported a similar pattern using a standardized 3T SWI protocol, with swallow tail sign loss occurring in 80% of DLB participants compared with 10% of participants with subjective memory complaints and less frequently in AD and frontotemporal dementia. Collectively, these findings provide complementary evidence of susceptibility-sensitive nigral abnormalities in DLB.

Finally, a single study employing the R2* metric evaluated individuals with prodromal DLB, defined as an early functional phase of the disease (Chabran et al., 2022). Decreased R2* signal was observed in the bilateral superior frontal cortex, left superior temporal cortex, right inferior longitudinal fasciculus, right lingual/fusiform gyrus, and the bilateral brainstem (midbrain) (Chabran et al., 2022). However, thalamic R2* values were positively correlated with cognitive fluctuation scores among DLB participants. Given differences in biophysical sensitivity between QSM and R2*, as well as the early disease stage examined, these findings require replication but nonetheless suggest that susceptibility measures remain associated with clinical features of disease.

#### 3.5.1 Strengths and limitations of *in vivo* studies

Strengths include the use of non-invasive MRI-based susceptibility measures, enabling regional assessment of brain tissue across cortical and subcortical structures *in vivo* (Wang and Liu, 2015). Several studies incorporated moderate to large sample sizes, well characterized clinical cohorts, and standardized diagnostic criteria. Advanced analytic approaches were commonly applied, including voxel wise and region of interest analyses, multivariate modeling, and in some cases correction for multiple comparisons. One study additionally applied a supervised machine learning classifier to regional QSM values, successfully distinguishing DLB patients by their earliest clinical presentation (Capogna et al., 2026), illustrating the potential for susceptibility-based data to inform patient stratification beyond simple group-level comparisons. Multi echo acquisition, high field strength imaging, and careful segmentation approaches enhanced measurement quality in several investigations. Some studies also accounted for relevant confounders such as age, sex, atrophy, and medication use, and extended analyses to clinical outcomes, including cognitive and motor measures.

Limitations include the indirect nature of susceptibility-based measures. QSM and R2* are related but distinct MRI-derived measures of tissue magnetic properties (Wang and Liu, 2015; Cheng et al., 2020; Verma et al., 2022). QSM estimates magnetic susceptibility, for which iron is a major contributor in many brain regions, whereas R2* is influenced by additional tissue characteristics, including myelin, microstructure, and extracellular water (Schweser et al., 2011; Hametner et al., 2018). Consequently, QSM is generally considered a more specific and interpretable approximation of regional brain iron burden than R2*, with validation across multiple neurodegenerative conditions, including PD (Du et al., 2016; Cheng et al., 2020; Saikiran and Priyanka, 2020; Verma et al., 2022; Dong et al., 2025). SWI-based visual or semi-quantitative assessment of the swallow tail sign represents a further methodological distinction. Unlike QSM, which provides a quantitative estimate of tissue magnetic susceptibility, SWI studies primarily assessed the appearance or signal intensity of nigrosome-1 using visual ratings or normalized signal-intensity measures. The reproducibility of these assessments varied across studies, with interrater agreement ranging from moderate to high (Kamagata et al., 2017; Shams et al., 2017; Rizzo et al., 2018). This rater dependence, together with differences in field strength, acquisition parameters, and image resolution, may limit comparability across SWI studies (Vaccarino et al., 2025). Moreover, loss of the swallow tail sign is not a direct measure of iron burden and may reflect multiple underlying processes, including alterations in iron storage, neuromelanin, and dopaminergic neuronal integrity.

Modality-specific differences in findings across the reviewed studies likely reflect underlying differences in biological sensitivity. Decreased R2* signal in prodromal DLB, for example, may reflect early myelin loss or microstructural disruption rather than reduced iron *per se* (Chabran et al., 2022), and need not contradict increased magnetic susceptibility in QSM observed in more established disease. However, both QSM and R2* modalities reflect composite magnetic properties influenced by iron, myelin, calcium, and other tissue characteristics, attribution of signal changes specifically to iron remains limited (Schweser et al., 2011; Zhang et al., 2016; Hametner et al., 2018). Susceptibility estimates are further influenced by acquisition parameters, echo configuration, segmentation strategies, statical approaches (Schweser et al., 2011; Wang and Liu, 2015). All studies were cross-sectional, precluding assessment of temporal dynamics. Additional limitations include variability in cohort composition, incomplete control of demographic and clinical confounders, and reliance in some cases on PD-derived clinical scales that may not fully capture LBD-specific features (McKeith et al., 2017). Comparator group composition also varied across studies; two of the three SWI studies compared DLB against symptomatic but non-demented groups (subjective cognitive impairment or subjective memory complaints) rather than confirmed healthy controls (Shams et al., 2017; Rizzo et al., 2018). This variability in comparator populations likely contributes to the range of diagnostic performance observed across SWI studies. Findings were also not uniform across all QSM studies; one investigation found no significant group differences in susceptibility across several subcortical regions, including the SN, when comparing DLB, AD, and healthy controls (Musaeus et al., 2026). Notably, the study included no female LBD participants, limiting the generalizability of its findings and underscoring the need to consider differences in cohort composition, brain regions examined, and acquisition protocols when interpreting variability across QSM studies.

*In vivo* neuroimaging provides the most consistent evidence of brain iron-sensitive susceptibility alterations in LBD, with most QSM studies demonstrating increased susceptibility across regions and disease stages, although this finding is not universal. SWI provides complementary evidence of susceptibility-sensitive abnormalities in the SN, with studies generally reporting loss or attenuation of the dorsolateral nigral hyperintensity (“swallow tail sign”) in DLB which may be easier to implement in a healthcare setting. However, unlike QSM, SWI-based assessment of the swallow tail sign does not directly quantify tissue magnetic susceptibility and may reflect multiple underlying pathological processes. In contrast, R2* findings remain limited to a single prodromal study and require further validation, although observed associations with clinical features suggest potential relevance in early disease.

### 3.6 Cross-compartment patterns and limitations

Across peripheral, postmortem, and *in vivo* brain studies, a uniform pattern of iron-related alteration in LBD was not observed. This variability may reflect differences in biological compartment and measurement approach, consistent with the complex regulation of iron across peripheral and central compartments (Chifman et al., 2014; Ward et al., 2014). Brain-based measures show the most directionally consistent evidence, though not without exception, whereas peripheral and CSF studies remain variable, a pattern consistent with the distinct regulatory biology of each compartment rather than methodological failure.

No included study directly evaluated relationships across peripheral, CSF, and brain-based measures within the same cohort, limiting the ability to define cross-compartment dynamics. Whether peripheral measures reflect lag, or are independent of central iron dysregulation in LBD remains an open question (Moos and Morgan, 2000; Zheng and Monnot, 2012; Garcia-Casal et al., 2021), and the compartment-dependent pattern observed here, while biologically grounded, remains inferential without within-cohort cross-compartment data.

Additional methodological factors further limit cross-study comparability. Disease stage varied considerably across cohorts, ranging from prodromal and early-stage disease to advanced dementia, with inconsistent reporting of disease duration and severity. Some studies classified participants based on neuropathological evidence of Lewy body pathology rather than clinical dementia criteria, capturing stages that are not directly comparable to clinically defined LBD. Clinical phenotype may represent an additional source of heterogeneity. Although Capogna et al. (2026) found no significant regional QSM differences between early cognitive/psychiatric and RBD/motor-predominant DLB phenotypes in univariate analyses, multivariate analysis distinguished the two groups based on a spatial pattern of QSM alterations across cortical and subcortical regions. This finding suggests that variation in iron-sensitive susceptibility within DLB may reflect distributed patterns associated with clinical phenotype rather than isolated regional differences. Sex-stratified analyses were absent from the available literature despite known sex differences in both iron biology and LBD clinical expression (Fairweather-Tait et al., 2014; Chiu et al., 2023; Bayram, 2025). Together, these differences in cohort composition and measurement approach limit cross-study comparability and make it difficult to characterize how iron-related measures evolve across the disease course, but present opportunities for future research.

## 4 Discussion

This scoping review synthesizes evidence from 24 studies across peripheral, postmortem, and *in vivo* compartments in LBD. Across heterogeneous cohorts and measurement approaches, two converging patterns emerged. Brain-based measures, particularly QSM and SWI, showed reproducible increases in iron-sensitive susceptibility linked to clinical symptoms and disease severity. Peripheral and CSF measures were variable and directionally inconsistent at the group level. These findings are consistent with a compartment-dependent model of iron dysregulation in LBD rather than a uniform disturbance of iron homeostasis (Chifman et al., 2014; Ward et al., 2014). Whether these compartmental changes are truly independent remains to be investigated, as no included study measured iron across all three compartments within the same cohort. Within the brain, the distributed regional pattern of iron-related alterations, extending beyond the SN, aligns with the known distribution of Lewy body pathology and cognitive and neuropsychiatric features that distinguish LBD from other synucleinopathies (Harding et al., 2002; Braak et al., 2003; Walker et al., 2015; McKeith et al., 2017).

Unlike PD, where iron accumulation within the SN is a consistent finding across postmortem and imaging studies (Wang et al., 2016), LBD evidence suggests a pattern that, while also consistently involving the SN, extends well beyond it to cortical, limbic, and subcortical regions. Despite shared synuclein pathology, these findings suggest that iron dysregulation in LBD may involve distributed networks rather than a primary SN-centered process. Postmortem studies identified increases in the cingulate cortex, frontal gray matter, motor cortex, hippocampus, and amygdala (Gu et al., 1998; Magaki et al., 2007; Scholefield et al., 2024). *In vivo* QSM studies similarly reported increased SN susceptibility (Chen et al., 2021; Diaz-Galvan et al., 2025; Capogna et al., 2026), alongside increases in superior frontal, medial temporal, superior temporal, and precentral cortices and hippocampus (Li et al., 2018; Chen et al., 2021; Bhome et al., 2025), with one study further extending this distributed pattern to the globus pallidus, thalamus, and brainstem (Capogna et al., 2026). Notably, Diaz-Galvan et al. (2025) directly linked antemortem QSM to postmortem confirmation of Lewy-related pathology within the same individuals, offering more direct support for QSM as a marker of the underlying pathological process. The regional distribution aligns with postmortem observations that Lewy body pathology is distributed across cortical and subcortical regions in varying degrees rather than confined to a single locus (Bayram et al., 2025; Sekiya et al., 2025; Gibson et al., 2026). The precise relationship between regional iron accumulation and Lewy body pathology distribution, however, remains to be defined.

Iron dysregulation in LBD also appears to change as the disease progresses. Chen et al. (2021) documented a progressive increase in SN susceptibility from healthy controls to prodromal synucleinopathy to probable DLB, suggesting that iron-related changes emerge early and evolve with disease progression. Although no associations with motor or cognitive measures were observed in that cohort, the progression of this gradient suggests that when and where iron-related changes occur may matter as much as whether they occur at all. Both biomarker development and clinical interpretation depend on understanding this timing and specificity.

The distinct regulatory biology of each compartment may limit the biomarker utility of peripheral iron measures. These measures may capture comorbid conditions, nutritional status, or systemic inflammation (Charlebois and Pantopoulos, 2023). Group-level peripheral findings in this review were heterogeneous and directionally inconsistent, potentially reflecting the influence of systemic processes on these measures. Despite absent group-level differences, peripheral measures were associated with disease symptoms in some individuals with LBD (Ayton et al., 2022; Ren et al., 2025), suggesting a complex relationship that may limit their utility as diagnostic proxies for central iron-related alterations. Serum transferrin followed a similar pattern, differing between PDD and non-demented PD and correlating with cognitive performance, despite no comparison to healthy controls being available (Guo et al., 2026). CSF measures, while anatomically proximal to the brain, also showed inconsistent findings and have not been directly demonstrated to reflect central iron status in LBD. Key modifiers of iron status, particularly inflammatory markers, were inconsistently assessed, further limiting interpretation (Infusino et al., 2012; Fairweather-Tait et al., 2014). Current evidence does not indicate that peripheral or CSF iron measures serve as standalone diagnostic indicators in LBD, though individual-level relevance warrants further investigation in longitudinal cohorts.

Among the available methodologies, *in vivo* neuroimaging provided the clearest evidence of brain iron alterations in LBD. Most QSM demonstrated increased magnetic susceptibility on QSM despite heterogeneity in cohort composition and disease stage, although this pattern was not universal (Chabran et al., 2022; Musaeus et al., 2026). Modality-specific differences between R2* and QSM likely reflect underlying differences in biological sensitivity or disease stage rather than true contradictions in the evidence base. Without histopathological validation, however, the degree to which observed susceptibility changes reflect iron biology rather than other tissue properties remains uncertain (Hametner et al., 2018). Importantly, clinicopathological validation demonstrated that histologically measured iron deposition was independently associated with QSM across all SN subregions, supporting iron as a major contributor to the susceptibility signal (Ono et al., 2026). However, tau burden also independently contributed to QSM in the ventromedial SN, predominantly through a pathway not mediated by iron, illustrating that elevated susceptibility cannot be attributed exclusively to tissue iron. Nonetheless, QSM values associated with disease severity, cognitive performance, and cognitive fluctuation scores, suggesting functional relevance beyond the imaging finding itself (Li et al., 2018; Chabran et al., 2022; Bhome et al., 2025). Regional QSM patterns have further distinguished DLB patients by early clinical phenotype, suggesting potential utility beyond group-level comparison (Capogna et al., 2026).

Beyond QSM, visual assessment of dorsolateral nigral hyperintensity, commonly termed the “swallow-tail sign,” on SWI has been investigated as a complementary and more clinically accessible imaging marker in DLB. Across the studies by Kamagata et al. (2017), Shams et al. (2017), and Rizzo et al. (2018), loss of this feature was more frequent in DLB than in other forms of cognitive impairment; a subsequent meta-analysis of these studies reported pooled sensitivity and specificity of 82% and 79% (Tseriotis et al., 2024). However, swallow-tail assessment primarily relies on visual characterization of nigrosome-1 appearance, and its biological basis is not specific to iron, potentially reflecting alterations in neuromelanin, iron handling, and dopaminergic neuronal integrity. SWI is nevertheless widely available on clinical MRI systems and does not require quantitative susceptibility reconstruction, potentially offering a pragmatic complement to QSM in clinical settings (Tseriotis et al., 2025; Vaccarino et al., 2025). Unlike postmortem approaches, MRI-based measures enable direct interrogation of brain tissue in living patients, offering a path toward clinical application. The reproducibility of QSM and SWI findings across independent cohorts and disease stages, combined with their associations with clinical features, positions iron-sensitive neuroimaging as a potential biomarker platform in LBD. Regulatory, academic, and industry stakeholders have identified improved biomarker development as a critical barrier to therapeutic progress in LBD (Agarwal et al., 2024). Longitudinal studies investigating the relationship between susceptibility signal and underlying tissue pathology are needed to establish iron-sensitive neuroimaging as a validated marker in LBD.

This distributed pattern of iron-related alterations maps onto the clinical phenotype of LBD and may help explain some of its most distinctive features. Fluctuating cognition and neuropsychiatric symptoms are core clinical features (McKeith et al., 2017) reflecting dysfunction within distributed cortical and subcortical networks (Gibson et al., 2025). Regional specificity related to symptoms is expected given LBD’s biological and clinical heterogeneity. Consistent with this possibility, Capogna et al. (2026) found that although individual regional QSM measures did not differ significantly between patients with early cognitive/psychiatric and early RBD/motor-predominant phenotypes, a multivariate pattern of cortical and subcortical QSM features distinguished the two groups. This suggests that clinically relevant susceptibility differences may be expressed as distributed spatial patterns rather than isolated regional abnormalities. Symptoms differ across subtypes, with greater early motor involvement in PDD than DLB (McKeith et al., 2017), therefore, cohort-level variation in regional susceptibility findings should be expected. Limbic and hippocampal involvement has been linked to neuropsychiatric features including visual hallucinations (Yao et al., 2014, 2016), while integrative hubs such as the thalamus may contribute to both motor and non-motor manifestations (Li et al., 2018). Associations between susceptibility measures and clinical symptoms suggest these regional changes reflect underlying disease processes, a distinction particularly relevant to the cognitive and neuropsychiatric features that differentiate LBD from other synucleinopathies (Li et al., 2018; Chabran et al., 2022; Bhome et al., 2025). Subtype-stratified and symptom-focused analyses are therefore warranted in future studies.

Stage-specific patterns of iron-related changes observed in LBD (Chen et al., 2021) have direct clinical implications that extend beyond established disease. A prodromal detection window may therefore exist, with meaningful clinical significance given LBD’s diagnostic delays. In prodromal DLB, thalamic susceptibility was associated with cognitive fluctuation scores, suggesting that iron-sensitive signals retain clinical relevance even at early disease stages (Chabran et al., 2022). However, the literature is almost entirely cross-sectional, and whether iron-related changes precede, accompany, or follow symptom emergence remains unknown. The potential suggested by these findings remains to be tested in longitudinal cohorts, a gap that is not merely methodological but clinically urgent given the scale of diagnostic delay in LBD.

Sex differences in iron status were identified but not further explored in at least one included study (Karismaz et al., 2025), representing a clinically significant gap in the available evidence. Iron biology diverges by sex over the lifespan: lifelong female menstrual iron loss and age-related male iron accumulation create fundamentally different iron profiles, directly affecting biomarker interpretation (Fairweather-Tait et al., 2014). Females also face higher misdiagnosis rates in LBD and may differ in Lewy body pathology distribution and clinical presentation, making sex a potentially important modifier on both iron biology and diagnostic accuracy (Chiu et al., 2023; Bayram, 2025; Bayram et al., 2025). Sex-stratified analysis in future biomarker studies is scientifically warranted and represent an unaddressed dimension of diagnostic equity.

LBD remains frequently underdiagnosed and misclassified (Armstrong et al., 2024; Palushaj et al., 2025), with prolonged diagnostic delays that limit opportunities for early intervention and appropriate management (O’Brien et al., 2021; Armstrong and Barnes, 2024). The consequences of diagnostic barriers in LBD extend well beyond the individual patient. Compared with other dementias, LBD drives higher healthcare utilization, greater caregiver burden, and increased likelihood of long-term care placement (Wyman-Chick et al., 2024), contributing to substantial economic burden on patients, caregivers, and health systems (Desai et al., 2022). Fluctuating cognition and neuropsychiatric symptoms, major drivers of caregiver strain and institutionalization (Armstrong and Barnes, 2024; Wyman-Chick et al., 2024), are associated with reduced survival (McCarter et al., 2025). These features may reflect dysfunction within cortical networks (Gibson et al., 2025), the same distributed regions implicated by iron-related alterations in this review, suggesting iron dysregulation may contribute to the features of LBD that carry the greatest clinical and economic cost. Reliable biomarkers are a critical unmet need, and their development must be accompanied by efforts to ensure patients reach clinical evaluation early enough to benefit (Galvin, 2024; Isaacson et al., 2024). Importantly, the QSM studies reviewed here primarily demonstrate group-level differences and associations with clinical features rather than individual-patient diagnostic accuracy. Based on the current evidence, QSM is therefore best considered a candidate imaging biomarker in LBD rather than a clinically validated diagnostic biomarker.

Limitations of the review process itself should also be considered. Searches were restricted to two databases and did not include databases such as Embase or Scopus, and the exclusion of gray literature and non-English records may have introduced publication bias. These constraints should be considered when interpreting the breadth of evidence presented here.

## 5 Conclusion

This review suggests that iron-related alterations in LBD differ across biological compartments rather than presenting a uniform pattern. Brain-based measures, particularly QSM, provide the most consistent evidence of iron-sensitive alterations distributed beyond the SN, complemented by visual SWI-based assessment as a more clinically accessible marker of nigrosome-1 integrity. Peripheral and CSF measures showed inconsistent group-level differences, and based on current evidence, do not appear to reliably track central iron status. Together, these findings suggest a central iron-related biological signal, partially obscured by methodological limitations, with direct relevance to the cognitive and neuropsychiatric features that distinguish LBD clinically. The considerable clinical and economic burden of LBD, compounded by its frequent underdiagnosis, underscores the need for improved biomarker characterization. Longitudinal studies integrating measurements across biological compartments, with standardized diagnostic criteria accounting for LBD subtypes, are needed to clarify the temporal dynamics of iron dysregulation and advance diagnosis, disease monitoring, and therapeutic development.
