# Supplementary material for: Iron-related biomarkers and Lewy body dementia: a scoping review
Source: Front Aging Neurosci. 2026 Oct 8;18:1900516. doi: 10.3389/fnagi.2026.1900516 (PMC13687944; doi:10.3389/fnagi.2026.1900516)
Supplement: Supplementary file 1 [file Supplementary_file_1.docx]

**Supplementary material 1.**

**Supplementary Methods: Database Search Strategies**

The following section provides the full database-specific search strategies used to identify studies for inclusion in this review. Searches were conducted in PubMed and Web of Science without date restrictions using controlled vocabulary terms and keyword combinations related to Lewy body dementia and iron-related biomarkers**.**

a) **PubMed search terms for full primary LBD_IRON: PubMed**

*(*

*"Lewy Body Disease"[Mesh]*

*OR "Lewy body dementia"[tiab]*

*OR "dementia with Lewy bodies"[tiab]*

*OR "Parkinson disease dementia"[tiab]*

*OR "Parkinson's disease dementia"[tiab]*

*OR DLB[tiab]*

*)*

*AND*

*(*

*ferritin[tiab]*

*OR transferrin[tiab]*

*OR "transferrin saturation"[tiab]*

*OR TSAT[tiab]*

*OR TIBC[tiab]*

*OR IBC[tiab]*

*OR hepcidin[tiab]*

*OR sTfR[tiab]*

*OR "iron deficiency"[tiab]*

*OR iron deficien**

*OR "iron overload"[tiab]*

*OR "brain iron"[tiab]*

*OR "iron deposition"[tiab]*

*OR "iron accumulation"[tiab]*

*OR siderosis[tiab]*

*OR hemosiderin[tiab]*

*OR QSM[tiab]*

*OR "quantitative susceptibility mapping"[tiab]*

*OR SWI[tiab]*

*OR "susceptibility-weighted imaging"[tiab]*

*OR "susceptibility weighted imaging"[tiab]*

*OR "R2 star"[tiab]*

*OR "R2-star"[tiab]*

*OR "nigrosome-1"[tiab]*

*OR "nigrosome 1"[tiab]*

*OR "dorsolateral nigral hyperintensity"[tiab])*

*NOT*

*(*

*"Review"[pt]*

*OR "Meta-Analysis"[pt]*

*)*

b) **Web of Science (EBSCO host)** **s*earch terms for full primary LBD_IRON***

("Lewy Body Disease"
OR "Lewy body dementia"
OR "dementia with Lewy bodies"
OR "Parkinson disease dementia"
OR "Parkinson's disease dementia"
OR DLB)
AND
(ferritin
OR transferrin
OR "transferrin saturation"
OR TSAT
OR TIBC
OR IBC
OR hepcidin
OR sTfR
OR "iron deficiency"
OR "iron deficien*"
OR "iron overload"
OR "brain iron"
OR "iron deposition"
OR "iron accumulation"
OR siderosis
OR hemosiderin
OR QSM
OR "quantitative susceptibility mapping"
OR SWI
OR "susceptibility-weighted imaging"
OR "susceptibility weighted imaging"
OR "R2 star"
OR "R2-star"
OR "nigrosome-1"
OR "nigrosome 1"
OR "dorsolateral nigral hyperintensity")

*+ manually excluded Review Articles and meta-analyses*
